# How safe are intra-articular corticosteroid injections to the hip?

**DOI:** 10.1186/s12891-023-06766-3

**Published:** 2023-08-22

**Authors:** Laura Elisa Streck, Sebastian Braun, Kimi Spilo, Cosima Sue Boettner, Marco Brenneis, Friedrich Boettner

**Affiliations:** 1 Hospital for Special Surgery, 535 East 70th Street, New York, NY 10021 USA; 2 Department of Orthopedics (Friedrichsheim), University Hospital Frankfurt, Goethe University, 60528 Frankfurt/Main, Germany

**Keywords:** Rapidly progressive osteoarthritis, Rapid destructive osteoarthritis, Intra-articular injection, Corticosteroid, Triamcinolone, Periprosthetic joint infection

## Abstract

**Background:**

Intra-articular corticosteroid injections (ICSI) are an effective symptomatic treatment for osteoarthritis of the hip. However, the safety of ICSI has been questioned and a relatively high risk for septic arthritis, rapidly progressive osteoarthritis (RPIO) and periprosthetic joint infections (PJI) in patients undergoing subsequent total hip arthroplasty (THA) have been suggested.

**Methods:**

This is a retrospective evaluation of 682 hips that underwent ICSI with 40 mg of Triamcinolone for primary osteoarthritis of the hip. All ICSI were performed using sterile techniques, the number of ICSI in each hip and the cumulative corticosteroid dosage were assessed. Pre- and post-injection radiographs were compared to identify cases with RPIO. Native joint septic arthritis, surgical site infections and PJI were identified by chart review.

**Results:**

4 hips (0.6%) developed RPIO 2–4 months following ICSI. The cumulative Triamcinolone dose was not associated with the development of RPIO (p = 0.281). 1 case was diagnosed with septic arthritis and treated with staged THA, there were no signs of infection at a 5 years follow-up. 483 hips (75.7%) underwent THA, including 199 hips with THA less than 3 months following ICSI and 181 hips with > 1 ICSI prior to THA. There were 3 superficial surgical site infections/wound dehiscence and no PJI.

**Conclusion:**

The rate of RPIO was 0.6%. The current findings suggest that if ICSI is performed under sterile conditions, the risk for septic arthritis or PJI following THA, even in patients with multiple ICSI or ICSI within 3 months prior to surgery, is minimal.

## Background

Intraarticular corticosteroid injections (ICSI) can relieve pain in patients with symptomatic osteoarthritis (OA) for weeks [1, 2]. A recent randomized controlled trial showed superior pain improvement following ICSI compared to treatment without injection over a period of 6 months [3]. Atchia et al. compared ICSI to saline-injection and treatment without injection and found ICSI to be the only treatment resulting in significant improvement in both pain and function [4]. Qvistgaards et al. reported a better effect on pain while walking following ICSI compared to saline injection [5]. ICSI may therefore allow to delay hip replacement [6]. However, there is an ongoing discussion regarding the safety of ICSI. There are three concerns including (1) septic arthritis, (2) rapidly progressive idiopathic osteoarthritis (RPIO) and (3) periprosthetic joint infections (PJI) following later total hip arthroplasty.

Multiple case reports on septic arthritis following ICSI have been published [7–10]. The main risk factors are avoidable and include improper sterile techniques and untrained physicians [11]. Another concern raised is the acceleration of OA progression and onset of RPIO [12]. Some studies reported on RPIO rates of 2.8–21% following ICSI [13–16]. In contrast, Abraham et al. found no differences in OA progression or femoral head collapse between patients with and without ICSI [17]. The methods and interpretation of studies on RPIO have been criticized [18]. It has been suggested that ICSI increase the risk of PJI for subsequent THA. Some studies found higher PJI rates if ICSI was performed within 3 months prior to total hip arthroplasty (THA) [19–22]. It has also been suggested that higher corticosteroid doses and multiple injections may result in a higher risk of PJI [23, 24]. However, other studies could not confirm these findings [25, 26].

While the discussion on the safety of intraarticular injections is ongoing, the American College of Rheumatology/Arthritis Foundation strongly recommends ICSI as one pillar in the non-surgical treatment of OA. To ensure accurate drug delivery into the joint, they also strongly recommend performing the procedure under ultrasound guidance [27].

The current study aims to answer the following research questions: (1) What is the frequency of RPIO following ICSI?, (2) Is there a correlation between the cumulative injected corticosteroid dose and onset of RPIO?, (3) What is the frequency of septic arthritis following ICSI?, (4) What is the frequency of PJI in hips that underwent ICSI prior to hip replacement?

## Methods

This is a retrospective evaluation of patients who underwent one or more ICSI for symptomatic primary OA of the hip. 1083 hips were injected between January 2015 and March 2020 by the senior author. 29 cases were excluded for indication other than primary OA (posttraumatic osteoarthritis, avascular necrosis), 372 cases were excluded as not all required radiographs (see below) were available. 682 hips in 638 patients were eligible for evaluation.

### Injections

All ICSI were performed following two times skin disinfection with Chlorhexidine/Isopropyl alcohol (Prevantics, PDI, Woodcliff Lake, NJ, USA), the surgeon used sterile gloves and techniques for the procedure. Each injection contained 1 cc Kenalog-40 (Bristol-Myers Squibb, Princeton, NJ, USA), 4 cc of Lidocaine 1%, and 4 cc of Marcaine 0.25% (equaling 40 mg Triamcinolone acetonide, 40 mg Lidocaine and 1 mg Marcaine). There were no standardized intervals between the injections, timing of the repeat injection depended on individual patients’ symptoms, however, injections were not repeated within 3 months. The number of ICSI in each hip and the cumulative corticosteroid dosage were assessed.

### Radiographic assessment

Antero-posterior and lateral radiographs of the hip prior to injection were compared to radiographs at least 6 months after the last injection. If patients underwent hip replacement less than 6 months following the last injection, preoperative radiographs were assessed. Antero-posterior radiographs were performed weight-bearing, lateral radiographs were performed non-weight-bearing in a supine position. Stage of osteoarthritis was defined according to the KellgrenLawrence Score in stages 0–4 [28, 29]. Prearthritic joint space width was assumed to be 4.5 mm lateral and 4 mm medial [30]. RPIO was diagnosed if (1) hips with > 50% joint space width at the initial radiograph showed progression to bone-to-bone OA or (2) hips with < 50% joint space width at the initial radiograph showed > 3 mm bone loss of the femoral head. All radiographs were initially reviewed by the first and the second author, respectively. Inter-rater reliability was good (κ = 0.819). Cases in which the raters disagreed and/or diagnosed RPIO were additionally reviewed by the senior author. The radiographic assessment and measurements were obtained in SECTRA PACS software package IDS7 (Sectra AB, Linkoeping, Sweden).

### Complications

The following complications were identified by retrospective chart review: (1) all cases: septic arthritis of the native joint and in cases that underwent subsequent THA within 2 years following ICSI: (2) surgical site infections (SSI) and (3) PJI.

### Statistical methods

Descriptive statistics were performed to describe means, range and standard deviation (SD) for all variables. The association between Triamcinolone dosage and RPIO was assessed by binary logistic regression analysis. Cohen’s Kappa (κ) was calculated to test inter-rater reliability. Statistical analysis was performed for a 95% confidence interval (CI), significance level was set at α = 0.050. Statistical analysis was performed with SPSS Statistics 22 (IBM, Armonk, New York, USA).

The study has been approved by the author’s institutional review board (IRB Number 2022 − 0601).

## Results

### Demographics and stage of osteoarthritis at the time of injection

The right hip was affected in 52.9%, the left hip in 47.1% of cases. 54.3% of the cases were female, 45.7% were male. The mean body-mass-index (BMI) was 28.1 kg/m² (range 17.3–46.2 kg/m², SD 5.6). The mean age at the first ICSI was 61 years (range 23–89 years, SD 10). Information on comorbidities can be found below under “Patients with special conditions”. At the time of the first injection stage of osteoarthritis according to the Kellgren-Lawrence score was grade 0 in 0.4% (3 hips), grade 1 in 4.5% (31 hips), grade 2 in 23.3% (158 hips), grade 3 in 50.7% (346 hips), and grade 4 in 21.1% (144 hips).

### Injections

The mean number of ICSI to the hip was 1.9 (range 1–9, SD 1.6), the mean cumulative Triamcinolone dose was 75 mg (range 40-360 mg, SD 62 mg). Details on number of ICSI and Triamcinolone doses are presented in Table 1. The mean time period between the first and last ICSI was 19 months (range 0-118 months, SD 21).

**Table 1:** Number and percentage of all hips that underwent a certain number of ICSI, and corresponding cumulative Triamcinolone doses. 79% of the hips were injected 1 or 2 times

Number of ICSI	Cumulative Triamcinolone dose [mg]	Number of hips	Percent of all hips [%]
1	40	424	62.2
2	80	113	16.5
**3**	**120**	**65**	**9.5**
**4**	**160**	**29**	**4.3**
**5**	**200**	**19**	**2.8**
**6**	**240**	**12**	**1.8**
**7**	**280**	**10**	**1.4**
**8**	**320**	**4**	**0.6**
**9**	**360**	**6**	**0.9**

### Rapidly progressive osteoarthritis and septic arthritis

**Fig. 1:**
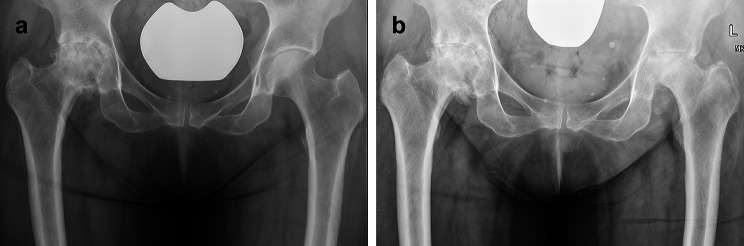
Radiographs of the pelvis of a 60-year-old female in antero-posterior view. (a) Radiograph prior to ICSI to the left hip: The left hip shows joint space narrowing and subchondral sclerosis. The right hip shows severe OA with multiple osteophytes, subchondral cysts, and joint space narrowing. Despite the radiographic findings, the right hip was clinically less symptomatic than the left hip. (b) Radiograph 4 months after ICSI to the left hip. There are no radiographic changes in the right hip. The left hip shows RPIO with bone-to-bone OA and severe bone loss of the femoral head

**Fig. 2:**
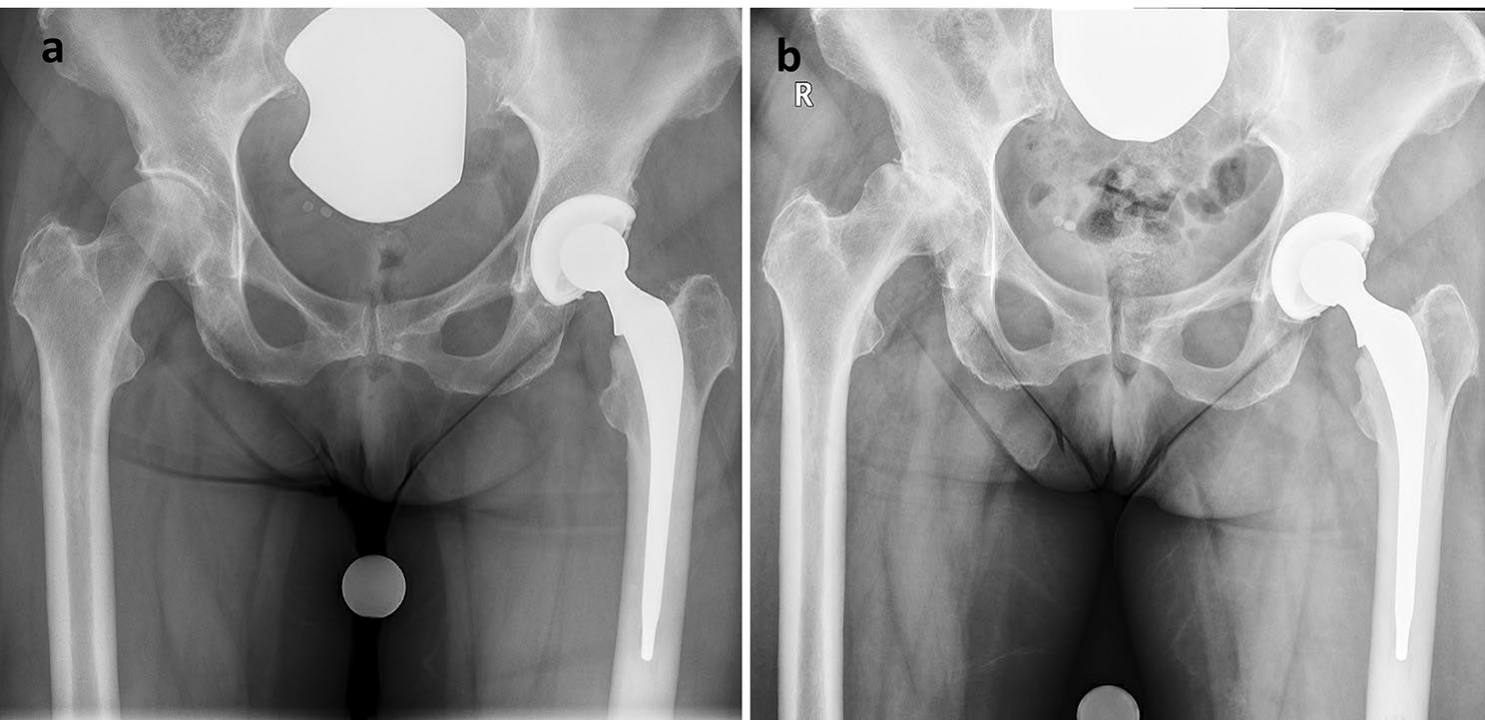
Radiographs of the pelvis of a 71-year-old female in antero-posterior view. (a) Radiograph prior to ICSI to the right hip: The right hip shows joint space narrowing, subchondral sclerosis and initial osteophytes. A total hip replacement with cemented stem is present on the left side, the arthroplasty is in correct position with no signs of loosening. (b) Radiograph 4 months after ICSI to the right hip. The left hip shows RPIO with bone-to-bone OA and severe bone loss and off-centered femoral head

389 hips (57.0%) underwent hip replacement less than 6 months following ICSI, in these cases, preoperative radiographs were evaluated. The mean time between last ICSI and radiographs in all other hips was 17 months (range 6–82 months, SD 13). A total of 4 hips (0.6%) were diagnosed with RPIO. The stage of Osteoarthritis at the time of injection in these cases was Kellgren-Lawrence grade 1 in 1 case, grade 2 in 1 case, and grade 3 in 2 cases. Figures 1 and 2 present radiographs prior- and post ICSI in cases diagnosed with RPIO. Time between ICSI and diagnosis of RPIO was 2 to 4 months, all underwent THA within 3–5 months following ICSI. There was no association between the cumulative Triamcinolone dose and the onset of RPIO (p = 0.281, CI: 0.995, 1.017).

### Total hip arthroplasty and postoperative complications

483 hips (75.7%) underwent THA 0-113 months following the first ICSI (mean 10 months, SD 14) and 0–45 months following the last ICSI (mean 4 months, SD 5). 199 hips underwent the last ICSI less than 3 months prior to THA and 181 hips had more than 1 ICSI prior to THA. Within a minimum follow-up of 2 years, there were no PJI. 3 cases were diagnosed with superficial (wound) dehiscence or SSI caused by Staphylococcus aureus. The time between ICSI and THA in these cases was 5 months (1 case) and 9 months (2 cases). All cases underwent superficial surgical irrigation and debridement without opening of the fascia joint itself. There were no signs of PJI at 2-year follow-up.

1 case was diagnosed with septic arthritis 2 months following ICSI, culture from preoperative diagnostic aspiration yielded growth of Neisseria gonorrhea, intraoperative culture yielded growth of Staphylococcus hominis. The patient had a history of unclear fevers and hip pain for 9 months prior to ICSI, he underwent diagnostic aspiration which resulted in a dry tap at an outside hospital and was prescribed prednisolone for rheumatoid arthritis. After being diagnosed with septic arthritis, he underwent two-staged THA and showed no signs of PJI within a > 5-year follow-up.

### Patients with special conditions

Amongst the study collective, 23 cases were diagnosed with rheumatoid arthritis or psoriatic arthritis, 21 cases were diagnosed with diabetes mellitus and 4 cases with a malignant disease (lymphoma, lung cancer). 35 cases were diagnosed with osteoporosis. Of those, 16 were treated with either Vitamin D only (13 cases), Estradiol subcutaneous patches (1 case), Denosumab (1 case), or Teriparatide at the time of injection which has been switched to Romosozumab injections later on during the follow up period (1 case). None of these patients had RPIO, septic arthritis, SSI or PJI.

## Discussion

The value of ICSI in the symptomatic treatment of hip OA is under discussion. The *Osteoarthritis Research Society International* (OARSI) does not recommend ICSI in its 2019 guidelines for non-surgical treatment of hip OA, as it was only classified as Level 3 treatment (meaning 40–59% votes in favor of ICSI) [31]. Britain’s *National Institute for Health and Care Excellence* (NICE) guideline reports inconsistent benefits on improving quality of life and function with ICSI, altough they found no evidence for long-term benefit beyond 3 months. Based on the potential benefits and the committee’s expert opinion, they recommend ICSI if “other pharmacological treatments are ineffective or unsuitable, or to support therapeutic exercise”, furthermore, patients should be advised that ICSI only provides short-term pain relieve of 2–10 months. Due to the lack of evidence, they recommended further research on ICSI [32].

In contrast, the *American College of Rheumatology/Arthritis Foundation* strongly recommended ICSI for patients with hip OA in its guideline published in 2019 [27].

In a review of randomized controlled trials, McCabe et al. found that ICSI delivered short but clinically significant pain reduction and may lead to transient functional improvement. The effect overall seemed to have an early onset and then decrease after 1 week [33]. However, two of the included studies reported significant improvements in both pain and function 2 months following ICSI [4, 34]. A recent study, published by Tang et al. in 2021 found that injections resulted in an average 5.1 months delay of THA [6]. A randomized controlled trial, published in 2022 by Paskins et al. found significantly greater improvement in hip pain over six months for patients who underwent additional ultrasound-guided triamcinolone-lidocaine injections compared to only information and advice on exercise, activities, weight loss, footwear, walking aids and pain management [3].

The current study focused on potential adverse events and did not address the efficacy of ICSI in the symptomatic treatment of hip OA. 38% of the cases underwent more than one ICSI and 29% of the hips underwent THA within 3 months following the last ICSI. This may suggest that a single ICSI only provided short term symptomatic effect and is unlikely to significantly delay surgery. However, including the cases with multiple ICSI, the mean time between the first ICSI and THA was 10 months, and the maximum time was 113 months.

### RPIO

The etiology of RPIO is not fully understood yet. Subchondral insufficiency fractures of the femoral head resulting from osteopenia have been suggested to be an underlying cause [35]. More recent findings also suggest that inflammation in the synovium plays a role in the development of RPIO [36]. Especially inflammation and osteoclast activation due to activation of inflammasome signalizing in the synovium may lead to rapid bone destruction [37]. A time- and dose- dependent effect of corticosteroids on cartilage has been described, suggesting beneficial effects at low doses and negative effects at high doses [38].

The exact mechanisms of how corticosteroid and triamcinolone affect cartilage, bone and osteoarthritis are not fully understood. In osteoarthritic joints, activated macrophages express growth factors and cytokines that may lead to extracellular matrix degeneration, synovial fibrosis and pain. Furthermore, the expression of bone morphogenetic proteins from synoviocytes increases which then induces osteophyte formation [39]. Glucocorticoids can induce activation of another type of macrophages, those regulatory macrophages induce cascades leading to a decrease in inflammatory cytokines while increasing Interleukin-10 [40, 41]. This effect has also been shown for Triamcinolone [39]. Furthermore, intraarticular injections with triamcinolone have been found to prevent osteophyte formation in arthritic joints [39, 42]. However, the exact mechanism remained unclear and a study by Ferrao Blanco et al. found triamcinolone injections to increase osteophyte maturation [43]. On the negative side, it has also been reported that corticosteroid treatment induced chondrocyte apoptosis in cultures and an vivo model [44]. Mice with triamcinolone injected to their osteoarthritic knees showed more subchondral sclerosis than the control group [39]. On the contrary, intraarticular corticosteroids have been found to reduce cartilage destruction in posttraumatic osteoarthritis [45]. She et al. compared dextran sulfate-triamcinolone acetonide conjugate nanoparticle injections to normal saline in a mouse model and found that the treatment alleviated osteoarthritis; that glycosaminoglycans were more organized, the synovia showed less inflammatory cells and there was less degeneration of chondrocytes [46].

It has been suggested that ICSI are associated with an increased risk for RPIO as some authors reported relatively high rates of RPIO following ICSI [13–16]. However, it remained unclear whether this was causative or coincidental [16]. RPIO has been found to randomly occur in patients who did not have underlying diseases or did not undergo prior interventions [47]. Villoutreix et al. reported on patients that underwent intra-articular injections after being diagnosed with RPIO and suggested that ICSI did not accelerate the course of destruction of the hip [48]. Abraham et al. found no differences in OA progression or femoral head collapse between patients with and without ICSI [17].

In our study collective, the rate of RPIO following ICSI was 0.6% and lower than previously reported [13–16]. One reason for highly inconsistent findings in the literature are the differences in the definition of RPIO. Okike et al. reported a RPIO rate of 5.4% utilizing the classification published by Zazgyva et al., however, they did not report a specific time interval [13, 49]. Hess et al., reported a RPIO rate of 21%, and defined RPIO as loss of cartilage greater than 2 mm or 50% joint space narrowing over ≤ 12 months [16]. Our study also included cases whose longest radiographic follow-up was 6 months. However, all cases presenting with RPIO developed the condition not more than 4 months following ICSI and the mean radiographic follow-up time of patients who did not undergo THA within ≤ 6 months following ICSI was 17 months. Thus, it seems unlikely that a longer minimum follow-up would increase the rates of RPIO significantly. Several studies reporting higher rates of RPIO following ICSI include small case numbers [16, 50]. And some studies have been criticized for their methodology as it may not allow for inference of causality regarding the reported complications [12, 18]. Furthermore, various patient specific risk factors for RPIO following intra-articular injections, such as BMI, age or female gender, have been reported [51, 52]. The current data suggests a relative safety of ICSI regarding the onset of RPIO, even in joints with repeated injections.

### Septic arthritis

The incidence of septic arthritis has been reported to be about 4–10 per 10 000 per year [53]. There are several case reports of septic arthritis of the hip following intra-articular injections [7–10]. It has been suggested that the coring of dermis and epidermis into the joint may be the underlying pathogenesis for septic arthritis following intraarticular injections [51]. However, reported risk factors are procedures performed by non-specialized providers using inadequate sterile techniques, and are therefore preventable [11]. An outbreak of knee infections following intra-articular injections in New Jersey in 2017 could be attributed to inadequate practice such as preparing syringes 4 days prior to injection and handling products for injection outside of pharmacy conditions [54]. In our cohort, 1 in 682 hips was diagnosed with septic arthritis following ICSI. In this case, the culture from diagnostic aspiration yielded growth of Neisseria gonorrhea, and intraoperative tissue cultures yielded growth of Staphylococcus aureus. Both pathogens have been described as main causing organisms in septic arthritis [7, 11]. The patient had several risk factors such as a history of rheumatoid arthritis and oral prednisone intake (10 mg Prednisone/day at the time of ICSI) [51]. A prior diagnostic aspiration had been performed at an outside hospital and a history of joint pain with fever and chills was documented prior to the ICSI. Therefore, it cannot be ruled out nor proven whether the septic arthritis was caused by the ICSI or whether the patient developed and acute on chronic infection, that was preexisting. This case underlines the necessity of careful assessment of patient history and special caution in patients with multiple risk factors.

### Periprosthetic Joint infection

Amongst 483 hips that underwent THA, no PJIs occurred related to ICSI within a minimum 2-year follow-up. The one PJI reported in the study, had symptoms prior to ICSI and likely had a longer standing chronic infection. This suggests that ICSI are not associated with an increased risk for PJI. In contrast, some studies found a higher PJI risk following ICSI, especially if the injection was performed within a 3 months interval before THA [19, 20, 22, 55]. It has also been suggested by Chambers et al. that multiple-injected joints were associated with higher PJI rates than single-injected joints [23]. Furthermore, Forlenza et al. reported a dose-dependent increase in the risk for PJI if the injection was performed within 3 months prior to surgery [24].

None of the studies provided information on the underlying pathogenesis that could fully explain these findings. Kaspar et al. hypothesized that there are potential sources of contamination, such as the steroid, its depot vehicle, the local anesthetic or that complications result due to the invasiveness of the injection needle through the skin [55]. Schairer et al. suggested either direct inoculation of bacteria and/or a decreased immune response made the joint more susceptible to infection [21]. The later seems unlike since steroids are regularly used for periarticular injection cocktails during total joint replacements [56]. Gosal et al. hypothesized that patients that intraoperatively receive low-dose inoculation of bacteria would usually overcome this but may not do so in the presence of corticosteroids [57].

Our study collective included 199 hips (41.2%) that underwent the last ICSI less than 3 months prior to THA and 181 patients (37.4% of the THA) that underwent 2–9 injections with cumulative Triamcinolone-doses up to 360 mg prior to surgery. None of these hips developed a PJI. This is in line with Sankar et al. who reported on 40 hips that underwent ICSI 2–23 months prior to THA, no PJI occurred within a mean follow-up of 23 months [58].

A survey amongst British doctors by Charalambous et al. showed that there is a wide variation in the practice concerning aseptic techniques prior to intra-articular injections and the authors noted a trend away from complete aseptic technique [59]. Disregarding hematogenous infections, there needs to be an inoculation of bacteria for PJI to occur. Therefore, we consider sterile techniques during ICSI to be of paramount importance in order to prevent infections.

### Limitations

The current study has several limitations: (1) the study design was retrospective, thus the study design did not include a control group and RPIO rates in patients without injections are not known, (2) the case numbers of patients with potential risk factors for infection or RPIO, such as diabetes mellitus and rheumatoid arthritis, were low and did not allow for proper statistical analysis, (3) the study includes patients that underwent THA < 6 months following ICSI, this needs to be considered when interpreting the results for RPIO, however these cases provide important information regarding PJI.

## Conclusions

The rate of RPIO was 0.6% and therefore lower than previously reported. The current findings further suggest that if ICSI is performed under sterile conditions, the risk for septic arthritis or PJI following THA is minimal, even in patients with multiple ICSI and/or ICSI within 3 months prior to surgery.

## Data Availability

The datasets used and/or analyzed during the current study are available from the corresponding author on reasonable request.

## List of abbreviations

CI: confidence interval
ICSI: Intraarticular Corticosteroid Injection
OA: Osteoarthritis
PJI: Periprosthetic Joint Infection
RPIO: Rapidly Prgressive Idiopathic Osteoarthritis
SD: standard deviation
SSI: Surgical Site Infection
THA: Total Hip Arthroplasty
κ: Cohen’s Kappa
